# On the Role of Bioinformatics and Data Science in Industrial Microbiome Applications

**DOI:** 10.3389/fgene.2019.00721

**Published:** 2019-08-09

**Authors:** Bartholomeus van den Bogert, Jos Boekhorst, Walter Pirovano, Ali May

**Affiliations:** ^1^Research and Development Dept., BaseClear, Leiden, Netherlands; ^2^NIZO Food Research, Ede, Netherlands; ^3^Bioinformatics Dept., BaseClear, Leiden, Netherlands

**Keywords:** DNA sequencing, microbiome, industrial biotechnology, probiotics, 16S rRNA gene profiling, metagenomics, bioinformatics, data science

## Abstract

Advances in sequencing and computational biology have drastically increased our capability to explore the taxonomic and functional compositions of microbial communities that play crucial roles in industrial processes. Correspondingly, commercial interest has risen for applications where microbial communities make important contributions. These include food production, probiotics, cosmetics, and enzyme discovery. Other commercial applications include software that takes the user’s gut microbiome data as one of its inputs and outputs evidence-based, automated, and personalized diet recommendations for balanced blood sugar levels. These applications pose several bioinformatic and data science challenges that range from requiring strain-level resolution in community profiles to the integration of large datasets for predictive machine learning purposes. In this perspective, we provide our insights on such challenges by touching upon several industrial areas, and briefly discuss advances and future directions of bioinformatics and data science in microbiome research.

## Introduction

Microbial communities play important roles in industrial processes such as the production of food, beverages, probiotics, paper, and cleaning products (for a review, see Singh et al., 2016). It has become an industrial standard to study the taxonomic composition and functional capabilities of these microorganisms using marker gene (e.g. 16S rRNA) and shotgun metagenome sequencing for product development, optimization, and quality control (Costessi et al., 2018). In addition, data from other omics sources such as metatranscriptomics and metabolomics can be used in integrative studies to generate leads, for instance in enzyme discovery. Some of the questions asked in these microbiome studies are related to determining the efficacy of probiotics and require strain-level characterization of the community composition (McFarland et al., 2018). Other studies focus on assessing the capability of microbial communities to produce certain compounds and necessitate recovering bacterial genomes from complex (e.g. soil) microbiomes (Howe et al., 2014). Extending microbiome applications to the public for actionable results, for example, to control blood sugar levels, requires a combination of advanced computational methods from bioinformatics, data mining, and machine learning (Zeevi et al., 2015).

In this perspective, we give an overview of several industrial microbiome applications with their bioinformatic and data science challenges. In addition, we highlight some of the advances that have the potential to provide valuable insights into the challenges facing these applications. We conclude with sharing our view on the future directions and requirements of industrial microbiome applications in terms of their computational components.

## Current Applications and Products

### Dairy Starter Cultures

Microbial populations (e.g. of lactic acid bacteria) are used in a variety of food and beverage production processes including the manufacture of cheese, yoghurt, meat, and wine. Specifically, their role in taste and structure formation is essential, for instance during cheese ripening. These processes are governed by the presence or absence of strain-specific enzymes (Escobar-Zepeda et al., 2016). Studying such enzymes through strain isolation is often costly and time-consuming since culturing strain representatives is difficult due to laborious or unknown growth conditions (Lagier et al., 2016). Alternatively, these enzymes can be studied by metagenome sequencing, assembly, and annotation, for instance, in product optimization (De Filippis et al., 2017). In addition, metagenome assembly plays an important role in analyzing bacteriophage populations in cultures in terms of their abundance, diversity, and development (Muhammed et al., 2017), which is important not only in the prevention of phage infections that cause fermentation failures, but also for unlocking the potential of phages against food-borne pathogens (Fernández et al., 2017).

### Probiotics

Probiotics are microbes that are intended to benefit the host health when consumed in adequate amounts. Identification of novel probiotics is a laborious process that starts with constructing a strain library using a culturomics approach (Lagier et al., 2016). This is followed by *in vitro* and computational research on the obtained strains to functionally characterize them, for instance for their bile resistance and potential to survive the passage of the stomach. Each of these steps reduces the list of high-potential candidates that as a final step must pass regulatory offices such as the European Food Safety Authority (EFSA, FEEDAP et al., 2018). We believe that the findings from comparative studies of the gut microbiome that highlight associations between phenotypic traits such as inflammation (Andoh et al., 2012) and obesity (Kasai et al., 2015) and specific bacterial populations, when integrated with other sources like metabolomic, demographic, dietary, and lifestyle datasets, may allow automated (e.g. machine learning-based) identification of candidate probiotic strains and reduce the time and financial cost of probiotics screening.

Small differences in the gene content of otherwise genetically identical bacterial strains can lead to different phenotypes (Zeevi et al., 2019), which in return may result in different outcomes *in vivo*. Therefore, well-conducted clinical trials are necessary to prove that the probiotic candidate itself confers the health effect. To make sure that the observed effects are not elicited by other (closely related) organisms and can be ascribed solely to the consumed probiotic, metagenomic, and bioinformatic methods that enable strain-level identification and tracking of the studied probiotic strain are required. For instance, in the genus *Bifidobacterium*, genetic differences between different strains of the same species underlie differences in carbohydrate utilization profiles (Arboleya et al., 2018). As these phenotypic traits are important in the development of probiotics for infant nutrition, applying shotgun metagenomics instead of amplicon sequencing for strain-level characterization may have substantial advantages.

### Quality Control

Products like probiotics and dairy starter cultures contain live organisms that are either sold directly to consumers or used to manufacture consumer products. Next to the checks performed for raw materials, quality control of the end product is necessary to ensure the presence of correct strains and the absence of pathogens (Fenster et al., 2019). As mentioned above, microbial strains of the same species can have vastly different phenotypes, making strain-level identification in the quality control process crucial for recognizing possible contaminants (Huys et al., 2013). Traditional typing approaches such Random Amplification of Polymorphic DNA-PCR (RAPD-PCR) can be used for identifying single-strain probiotics contaminants, but require cultivation (Mohkam et al., 2016), making them unsuitable for high-throughput screening of products with complex communities (e.g. probiotics and dairy products). Whole-metagenome sequencing and analysis has the potential not only to circumvent these lengthy processes in providing strain-level information, but also to enable screening of undesired traits such as (spore) heat-resistance based on the presence of associated genes (Berendsen et al., 2016).

### Cosmetics

The cosmetics industry has a growing interest in studies that aim to explore the skin microbiome as a potential therapeutic target for disorders including acne, eczema, and *Malassezia* folliculitis (Wallen-Russell, 2019). Unfortunately, these studies are typically hampered by the low biomass of skin samples, where small contaminations (e.g. from adjacent skin or reagents) can easily lead to incorrect outcomes (Kong et al., 2017). Furthermore, the human skin microbiome is strongly subject-specific (Zeeuwen et al., 2012), making it difficult to determine the effect of skin products on the general population. While this opens a potential market for personalized skin products, it also raises the need for personal longitudinal studies, where statistical methods such as redundancy analysis and principle response curve (Van den Brink and Braak, 1999) help assess correlations between taxonomic or functional composition and sample characteristics (environmental variables). Furthermore, the data can be corrected for one of the variables, such as ‘subject’ so that the variance from that covariate is removed before the actual analysis is performed, which facilitates determining the effect of the treatment.

### Enzyme Discovery

A wide range of industrial enzymes, such as those used in the production of cleaning agents, laundry detergents, paper, and textile, have the continuous demand to become cheaper, greener, and more efficient. Among others, marine, soil, and lake microbiomes, with their extremely high and mainly uncharacterized biodiversity, constitute exciting functional mines not only in the search for new enzymes with such desired properties, but also for the discovery of novel enzymes that can catalyze challenging reactions (Popovic et al., 2015). A notable example of the latter is the recent discovery of two enzymes that enable the production of a renewable alternative to toluene, a petrochemical with a market of 29 million tons per year, from complex microbial communities that live in sewages and lakes (Beller et al., 2018).

Two main bioinformatic challenges in metagenomic enzyme discovery arise from the same fact that makes the chosen environment (e.g. soil) attractive in the first place: its high and uncharacterized biodiversity. The large number of different genomes in the environment and their highly skewed abundance distribution make it difficult to obtain contiguous and complete assemblies (Ayling et al., 2019), an outcome that negatively impacts gene prediction. The next challenge lies in functionally annotating the predicted genes, where commonly a high percentage of sequences are labeled as “hypothetical” or with unknown function.

### Microbiome-Based Health and Personalized Nutrition

Companies and citizen science projects such as MyMicroZoo^1^, Biovis^2^, and American Gut^3^ offer affordable microbiome analysis services to general consumers. While operationally their analyses are the same as those used for research, they must pay far more attention to the clarity of the results to ensure correct interpretations by the end-users even if the results are stated not to be interpreted as diagnosis. In practice, this means that the end-user should be guided through the (actionable) results with the help of trained healthcare professionals [e.g. dieticians and general practitioners (GPs)], who should take the limitations of a given analysis into account to prevent overinterpretation.

While basing health-related advice on published research findings is a good practice, the fact that most studies focus on a defined cohort and report “averaged” population trends makes it questionable whether results can be translated back to individuals. Such translations to the individual may be less complicated with function-based approaches through metagenomics as the ‘personalized’ effects are less pronounced in these datasets (Lloyd-Price et al., 2017). Nonetheless, the predictive value of a person’s gut microbiome for health was demonstrated by an inspirational study by Zeevi and colleagues (2015), which integrated blood parameters, dietary habits, anthropometrics, physical activity, and the gut microbiome data into a machine learning algorithm that predicted the post meal glycemic responses of the subjects. Ultimately, 72 taxonomic or functional features of the microbiome were included in the predictive model. This approach, validated further with another independent cohort, is now offered to the public by DayTwo^4^, which is a good example of how extensive datasets from scientific studies and data science can be combined in an industrial setting for providing customers with evidence-based health-related recommendations.

## Current Advances

### Metagenome Assembly, Binning, and Annotation

Metagenome assembly enables gene prediction, annotation, and abundance profiling, and therefore is an important computational step when studying the functional composition and capacity of microbiomes. Many (de Bruijn graph-based) metagenome assembly methods that differ in terms of their ease of use, scalability, running time, and memory requirement exist, making it important to carefully choose the one that serves the research question at hand the best (Van der Walt et al., 2017). For instance, in comparative studies with large cohorts where the impact of probiotics on the abundances of gene groups and pathways is analyzed, tools that are computationally less intensive, such as MEGAHIT (Li et al., 2015), are preferred. In contrast, studies with a low number of samples, such as those in enzyme discovery applications, can make use of assembly tools like metaSPAdes (Nurk et al., 2017) that include optimizations such as error correction but with a subsequent runtime trade-off. When higher read depth for assembling low abundance members or recovering full genomes is needed, data from (not too) different samples (e.g. dairy starter cultures) can be combined using co-assembly methods like crass (Dutilh et al., 2012) which also facilitates metagenomic comparison between samples. Finally, binning methods such as MetaBAT (Kang et al., 2015), MaxBin (Wu et al., 2014), and COCACOLA (Lu et al., 2017) facilitate extracting individual (draft) genomes from metagenome assemblies, which helps look at a specific organism in more detail e.g. in enzyme discovery applications where identifying the genome that encodes the target enzyme is important.

In a recent study of cow rumen microbiome, a valuable environment for biomass-degrading enzyme discovery, Stewart et al. (2018) showed that 90% of the proteins predicted to be involved in the studied mechanism (carbohydrate metabolism) did not have a good match in public databases. Such findings highlight the relatively large room for improvement in microbiome annotation.

### Hypothesis-Driven Functional Analyses

Exhaustively analyzing all functional aspects and querying all potential longitudinal and cross-sectional aspects of a microbiome dataset is generally considered a hopeless task. Even when computationally feasible, multiple testing issues lead to a severe reduction of the analysis power. Although approaches like the removal of collinear variables and validation of potential correlations in independent datasets can in part address these issues (Falony et al., 2016), delineating the relevant functional aspects is a big step in overcoming these limitations. Using a specific database to answer a particular hypothesis, such as in the case with certain enzyme classes or a set of enzymatic pathways, is such an approach. Examples of such databases and tools are Resfams (Gibson et al., 2015), dbCAN (Yin et al., 2012), and antiSMASH (Blin et al., 2017), focusing on antibiotic resistance, carbohydrate utilization, and secondary metabolite synthesis, respectively. Methods developed for the elucidation of gene function, such as the guilt by association approaches implemented in STRING (Szklarczyk et al., 2014), can be used to identify genes that are not directly flagged by comparison to specific functional datasets such as the ones described above, but have distribution patterns similar enough to genes that are represented in the reference set. A drawback of functional analyses that require protein sequences is the need for assembly and gene prediction, which can be computationally intensive as described above. Tools like HUMAnN2 (Franzosa et al., 2018) work directly with short-read data without requiring an assembly for profiling protein family abundance.

### Assembly-Independent Strain-Level Characterization

Probiotic members such as *Bifidobacterium longum* subsp. *longum* and *Bifidobacterium longum* subsp. *infantis*, which have two distinct phenotypes with relevant functional implications in infant nutrition (Underwood et al., 2015), differ only slightly in their16S rRNA gene sequences (Lawley et al., 2017). Such differences are lost in classical operational taxonomic unit (OTU) clustering-based taxonomic analyses. Novel methods like UNOISE2 (Edgar, 2016) and DADA2 (Callahan et al., 2016) circumvent clustering and apply sequence filtering steps, enabling distinguishing between sequences on a single-nucleotide level by grouping reads in amplicon sequence variants (ASVs). This has a great potential to improve the phylogenetic depth at which microbiome studies can be interpreted. Notable applications of these new algorithms provided new, sub-species level insights into oral (Mukherjee et al., 2018) and vaginal microbiomes (Callahan et al., 2017).

**Figure 1:**
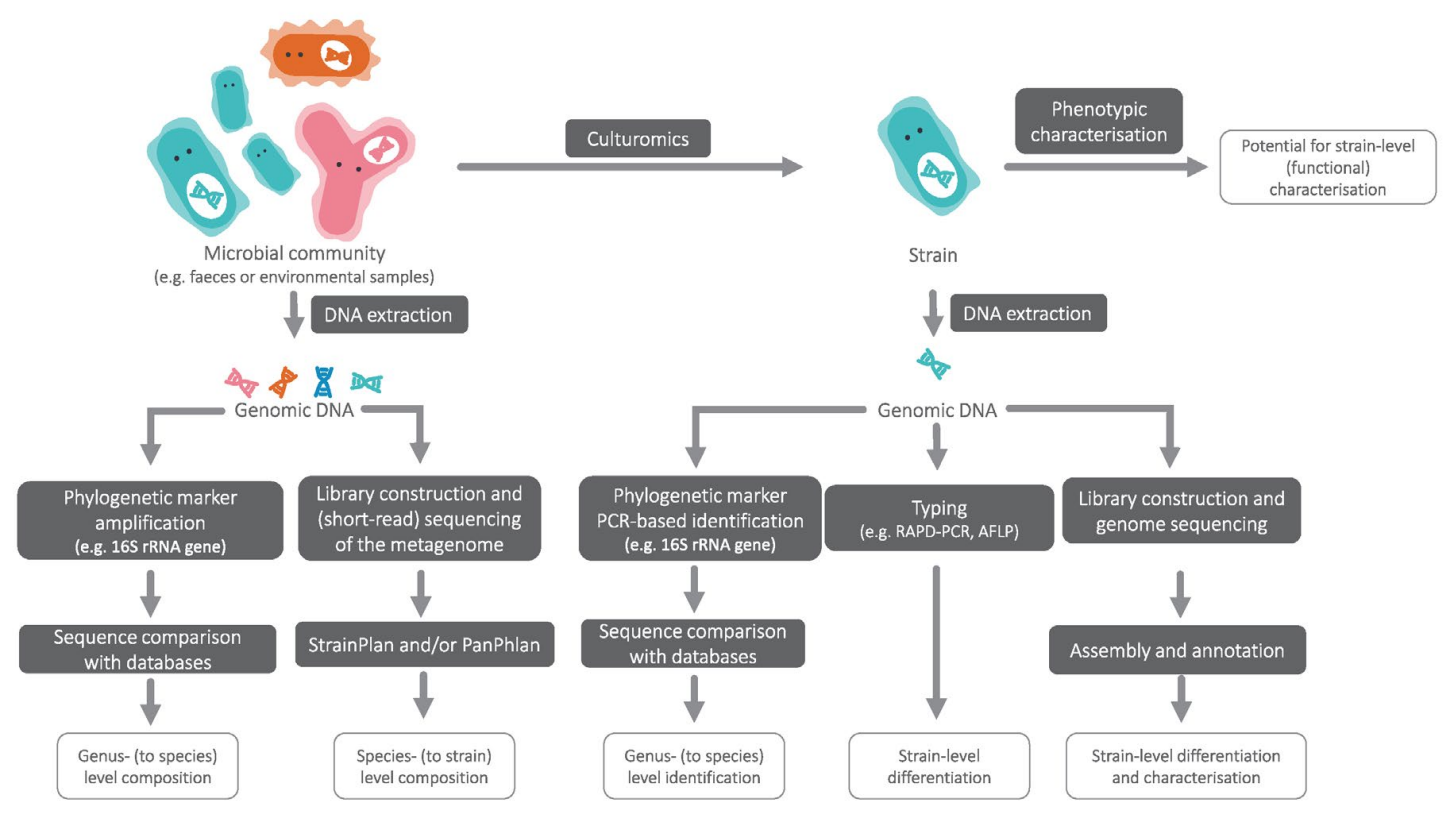
An overview of approaches to achieve taxonomic resolution at different levels.

In cases where multiple strains of a species of interest have identical 16S rRNA sequences, algorithms such as StrainPhlAn (Truong et al., 2017) and PanPhlAn (Scholz et al., 2016) enable strain-level analyses from shotgun metagenome datasets without the need for metagenome assembly (**Figure 1**). These methods open the possibility for routine compositional analyses to verify the presence of desired strains or identify potential pathogens in end products.

### Long-Read Sequencing and Other Advances

Although their use in microbiome studies is currently not common, long-read sequencing platforms Pacific Biosciences (PacBio) and Oxford Nanopore Technologies (ONT) offer exciting opportunities for several industrial applications mentioned above. For instance, circular consensus sequencing application by PacBio, which allows multiple reads generated from a circularized amplicon molecule to be bioinformatically combined into a high-quality, full-length (16S) sequence (Callahan et al., 2018), provides the necessary phylogenetic resolution for applications such as fermentation studies, which is unfeasible with short-read amplicon sequencing. The on-demand sequencing nature of ONT, on the other hand, seems suitable for quality control applications for detecting distinct pathogens, although the high error rate is limiting for accurate, strain-level detection.

Even with high dataset coverage and advanced methods, assemblies from short-read datasets commonly remain very fragmented, especially in samples from complex communities like soil. Soon, we expect the integration of long-read sequencing to be more common in assembly-oriented studies for obtaining full, chromosome-level microbial genomes. Correspondingly, we see potential in adapting hybrid assembly methods such as hybridSPAdes (Antipov et al., 2015) to enable their use with long- and short-read metagenome datasets. Other promising developments revolve around using barcoded short reads that have long-range information, such as those provided by 10x Genomics (http://10xgenomics.com), in microbiome research. We see the emergence of tailored bioinformatic methods such as the Athena assembler (Bishara et al., 2018), which uses barcode information in short-reads and improves the contiguity of metagenome assemblies.

### Machine Learning and Data Science

With decreasing sequencing costs, the size of datasets in microbiome studies and the depth of sequencing per sample have increased. This led to studies with higher statistical power, and consequently to the transition of OTU tables and functional profiles from end-goal deliverables into starting material for downstream analyses such as machine learning (ML) applications (Pasolli et al., 2016). Methods like random forests (RF) have been successfully used by many within a disease context, for instance, for accurately predicting irritable bowel syndrome (Saulnier et al., 2011) and bacterial vaginosis (Beck and Foster, 2014) based on taxonomic profiles (for a review, see LaPierre et al., 2019 and Qu et al., 2019). On the other hand, Sze and Schloss (2016) used 10 previously published obesity datasets and showed that RF ML models trained on one of the datasets and tested on the remaining nine had a median accuracy of only 56.68%, suggesting that i) the method may not be applicable for some diseases, or ii) the disease signal may be more apparent at the level of differentially expressed functions (gene transcripts) of the microbiome.

Industrial microbiome applications of ML include building classification models based on soil microbiome data for detecting oil sites (Miranda et al., 2019) and above-mentioned personalized health-related lifestyle (diet) recommendation services that are partly based on gut microbiome data. As mentioned in *Probiotics*, we expect dataset integration and ML to have an impact also on areas such as screening of novel probiotics. To meet the overall demand for user-friendly ML in microbiome research, software suites like QIIME 2 (Bokulich et al., 2018), MicrobiomeAnalyst (Dhariwal et al., 2017), and USEARCH (Edgar, 2010) started incorporating ML methods that can be used by researchers who aren’t necessarily trained as bioinformaticians.

## Conclusions and Outlook

The vast number of experimental and computational methods available for microbiome research have led to a broad collection of choices. While creation of guidelines and standardization for increased comparability and reproducibility is essential, achieving a global consensus in methods used remains a challenge. What constrains researchers to their current practices is mainly the laborious nature of adopting other (new) protocols, which may have an ironically detrimental effect on comparability between different studies, or even within studies that run over prolonged periods. Like Knight et al. (2018), we think that a primary objective of microbiome studies should be to standardize the documentation of used methods, tools, data formats, and data processing parameters, and to publish these “logs” next to the final results and interpretations. While complete disclosure is scientifically ideal, it raises commercial concerns for microbiome analysis providers like BaseClear^5^, NIZO food research^6^, Clinical Microbiomics^7^, Vedanta Biosciences^8^, and COSMOSID^9^, as it would mean releasing a substantial part of their, sometimes unique, intellectual property.

With reducing costs, we soon expect long-read sequencing technologies to be commonly used in microbiome studies, which will benefit from enhanced taxonomic resolution with full-length marker gene sequencing, as well as improved functional analyses thanks to more contiguous metagenome assemblies. Here, the focus in developments is likely to be on the translation of bioinformatic protocols already established for short reads to long-read versions, for instance in denoising and read classification approaches.

Other challenges relate to shotgun metagenome analyses in large studies, where expensive calculations used in *de novo* assembly and annotation may result in capacity issues. For companies that cannot afford large on-premise compute infrastructures, the cloud provides a flexible alternative, where know-how of cloud-computing becomes essential.

Finally, the rapid translation of microbiome research into important industrial applications in healthcare, energy, and food production will continue to stimulate collaborations between academic and industrial communities. We expect the role of bioinformatics and data science to become only more significant in this relationship.

## Author Contributions

All authors were involved in the writing and final preparation of the article.

## Conflict of Interest Statement

BB, WP, and AM work at BaseClear. JB works at NIZO Food Research.

## Footnotes

1 mymicrozoo.com

2 biovis-diagnostik.eu

3 humanfoodproject.com/americangut/

4 daytwo.com

5 baseclear.com

6 nizo.com

7 clinical-microbiomics.com

8 vedantabio.com

9 cosmosid.com
